# Non‐equivalence in old‐ and new‐flagellum daughter cells of a proliferative division in *Trypanosoma brucei*

**DOI:** 10.1111/mmi.14345

**Published:** 2019-07-25

**Authors:** Movin Abeywickrema, Hana Vachova, Helen Farr, Timm Mohr, Richard J. Wheeler, De‐Hua Lai, Sue Vaughan, Keith Gull, Jack D. Sunter, Vladimir Varga

**Affiliations:** ^1^ Sir William Dunn School of Pathology University of Oxford Oxford OX1 3RE UK; ^2^ Institute of Molecular Genetics of the Czech Academy of Sciences Vídeňská 1083 Prague 14220 Czech Republic; ^3^ Department of Biological and Medical Sciences Oxford Brookes University Oxford OX3 0BP UK; ^4^ Peter Medawar Building for Pathogen Research, Nuffield Department of Medicine University of Oxford Oxford OX1 3SY UK; ^5^ Center for Parasitic Organisms, State Key Laboratory of Biocontrol, School of Life Sciences Sun Yat‐Sen University Guangzhou 510275 P.R. China

## Abstract

Differentiation of *Trypanosoma brucei*, a flagellated protozoan parasite, between life cycle stages typically occurs through an asymmetric cell division process, producing two morphologically distinct daughter cells. Conversely, proliferative cell divisions produce two daughter cells, which look similar but are not identical. To examine in detail differences between the daughter cells of a proliferative division of procyclic *T. brucei* we used the recently identified constituents of the flagella connector. These segregate asymmetrically during cytokinesis allowing the new‐flagellum and the old‐flagellum daughters to be distinguished. We discovered that there are distinct morphological differences between the two daughters, with the new‐flagellum daughter in particular re‐modelling rapidly and extensively in early G1. This re‐modelling process involves an increase in cell body, flagellum and flagellum attachment zone length and is accompanied by architectural changes to the anterior cell end. The old‐flagellum daughter undergoes a different G1 re‐modelling, however, despite this there was no difference in G1 duration of their respective cell cycles. This work demonstrates that the two daughters of a proliferative division of *T. brucei* are non‐equivalent and enables more refined morphological analysis of mutant phenotypes. We suggest all proliferative divisions in *T. brucei* and related organisms will involve non‐equivalence.

## Introduction

Some cells have the ability to undergo proliferative, so‐called ‘symmetric’, cell divisions, generating daughters destined to the same fate, as well as asymmetric cell divisions, which generate daughter cells destined to different fates (Morrison and Kimble, 2006; Santoro *et al.*, 2016). An excellent example are mammary stem cells, which are able to proliferate as well as exploit asymmetric cell division during differentiation; this allows them to simultaneously generate differentiated cells and replenish themselves (Santoro *et al.*, 2016). The balancing of these two modes of division sustains tissue morphogenesis and homeostasis in such mammalian systems.

In certain organisms, particularly protists, the terms symmetric and asymmetric cell division have historically been used in a different context, referring to morphology of daughter cells post‐cytokinesis rather than their fates. Hence, in budding yeast two types of proliferative division have been described. First, asymmetric cell division of a single yeast cell produces a larger ‘mother’ cell inheriting the bud scar, and a smaller ‘daughter’ cell with the birth scar. This daughter spends longer time growing in G1 to reach a critical cell size for re‐entering the cell cycle (Hartwell and Unger, 1977). A budding yeast cell in a pseudohyphal form on the other hand divides into two visually similar cells by symmetric division; these daughter cells are of similar sizes and enter cell cycle synchronously (Kron *et al.*, 1994).

In a wider biological context, it has been recognized that although similar at the whole cell level, the daughter cells of such a ‘symmetric’ division may exhibit an element of asymmetry; this is thought to be due to certain segregated structures and organelles having a different history and hence being non‐identical. Examples include segregation of the mother and daughter centriole (Anderson and Stearns, 2009), yeast spindle pole bodies (Lengefeld and Barral, 2018), the primary cilium membrane (Paridaen *et al.*, 2013), and the new or old bacterial cell pole (Stewart *et al.*, 2005). These examples have been shown to correlate with, and contribute to, functional differences between the daughter cells, including asynchronous cilium formation and signalling (Anderson and Stearns, 2009), differences in growth rate and in propensity to ageing (Stewart *et al.*, 2005).

Here, we use the term ‘non‐equivalence’ to describe non‐identical products of proliferative divisions, in contrast to the use of asymmetry in differentiation divisions producing daughters with markedly different fates.

*Trypanosoma brucei* is a protozoan parasite of mammals causing Human African Trypanosomiasis (sleeping sickness) and Nagana in cattle. *T. brucei* is spread from host to host by tsetse flies. In their complex life cycle, trypanosomes undergo a defined sequence of proliferative and differentiation cell divisions, which generate life cycle stages adapted, biochemically and morphologically, for colonizing a particular environment (Matthews, 2005).

A trypanosome cell has a well‐defined morphology, which is determined by the microtubule‐based cytoskeleton underlying the plasma membrane. During the cell cycle microtubules elongate at their plus ends, which are located mainly in the zone at the posterior of the cell body. In the zone in the middle of the cell microtubules are nucleated alongside the existing ones and intercalate between them, leading to an increase in a cell's circumference. There is little microtubule polymerization in the zone at the cell anterior (Sherwin and Gull, 1989a; Wheeler *et al.*, 2013). Single copy organelles and structures, such as the flagellum, the flagellum attachment zone (FAZ), the basal body, and kinetoplast (mitochondrial DNA) are connected to this sub‐pellicular corset (Gull, 1999) and timing of their duplication is tightly linked to the cell cycle (Sherwin and Gull, 1989b; Robinson *et al.*, 1995).

As the microtubule‐based cytoskeleton does not disassemble during cytokinesis, it has to be re‐modelled to allow for its segregation into two daughter cells; hence, the cytoskeleton is inherited in a semi‐conservative way (Sherwin and Gull, 1989a). The flagellum and the FAZ, on the other hand, are inherited conservatively: one daughter cell inherits those constructed in one of previous cell cycles, while the other one those constructed in the current cell cycle (Sherwin and Gull, 1989b).

Due to these modes of structure and organelle inheritance in *T. brucei* and related parasites, such as *Trypanosoma cruzi* and *Trypanosoma vivax*, dramatic changes to cell morphology, such as reduction of cell and flagellar length (Sharma *et al.*, 2008; Kurup and Tarleton, 2014) and repositioning of cellular organelles during differentiation divisions (Rotureau *et al.*, 2012; Ooi *et al.*, 2016), are typically achieved by differential construction of the portion of the cell body that ultimately becomes the new‐flagellum daughter cell. This was also observed for *Trypanosoma congolense*; moreover, in this parasite re‐modelling of existing structures was recently also shown to be part of differentiation processes (Peacock *et al.*, 2018).

Given the conservative manner of specific organelle inheritance, the products of a proliferative *T. brucei* division are, despite having similar morphology, non‐equivalent. For clarity and convenience, we now refer to these as the OFD, old‐flagellum daughter and NFD, new‐flagellum daughter. Previous work has shown some differences between NFDs and OFDs (Farr and Gull, 2009; Wheeler *et al.*, 2013); however, we wanted to extend this and characterize in detail the daughters of a proliferative division of procyclic (tsetse midgut) stages of *T. brucei*. Procyclic cells can be readily cultured and hence serve as a model for the intrinsic cell biology of the parasite. Their cell cycle has been well characterised (Sherwin and Gull, 1989b; Woodward and Gull, 1990; Robinson *et al.*, 1995; Archer *et al.*, 2011; Benz *et al.*, 2017). In G1 phase, a cell has one nucleus, one kinetoplast and one flagellum (1F1K1N cell). In S phase, it starts to construct a second new flagellum (2F1K1N cell). In G2 phase the kinetoplast divides resulting in a 2F2K1N cell, followed by mitosis producing a 2F2K2N cell. Finally, cytokinesis occurs separating the 2F2K2N cell into two similar 1F1K1N cells—the NFD and the OFD.

Our study was facilitated by the recent identification of protein markers specific for each of the two daughter cells. These are constituents of the flagella connector (FC), the structure attaching the tip of the new flagellum to the side of the old flagellum during cell cycle of procyclic trypanosomes (Moreira‐Leite *et al.*, 2001). As the new flagellum elongates, the FC migrates along the old flagellum until it reaches a point approximately half way along the old flagellum (Davidge *et al.*, 2006). The FC is an asymmetric structure (Höög *et al.*, 2016), with specific proteins constituting its new and old flagellum zones (Varga *et al.*, 2017). Hence, FC severing, which occurs at variable points during cytokinesis (Briggs *et al*., 2004), produces two distinct remnants of the FC (from now on called the FCR—flagella connector remnant). The remnant at the tip of the new flagellum (N‐FCR) and the one approximately half way along the old flagellum (O‐FCR) contain specific protein constituents, which mark the respective daughter cells for limited time post cytokinesis (Varga *et al.*, 2017).

We report that elongation of the cell body, flagellum and the FAZ and cytoskeletal changes at the anterior cell end are required for the re‐modelling of the NFD. There is also re‐modelling of the OFD leading to a co‐incidence of cell organisation as both daughters proceed through the cell cycle. Re‐modelling is accomplished rapidly in the first portion of the G1 phase. Furthermore, we show that elongation of cytoskeletal structures and cytoskeletal rearrangements associated with re‐modelling of each daughter do not impact the average G1 phase duration.

## Results

### The antibody mAb62 recognises the flagella connector

**Figure 1:**
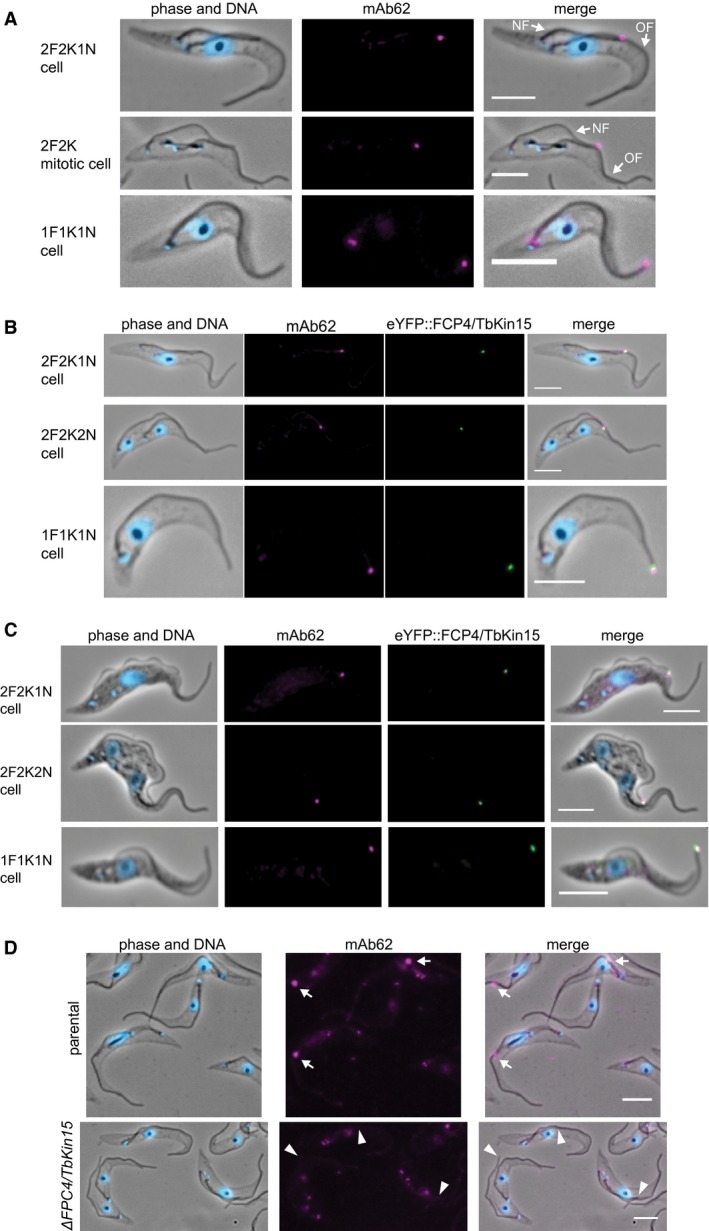
mAb62, a monoclonal antibody that recognizes the flagella connector and the flagella connector remnant in new‐flagellum daughter cells. Scale bars represent 5 µm. A. Micrographs of SMOxP9 detergent‐extracted cytoskeletons stained with mAb62 (magenta) and with DAPI stained DNA (blue). NF‐new flagellum constructed in the current cell cycle, OF‐old flagellum constructed in one of previous cell cycles. B. Micrographs of cytoskeletons of SMOxP9 cells expressing eYFP::FCP4/TbKin15 (green) stained with Ab62 (magenta) and the DNA stained with DAPI (blue). C. mAb62 (magenta) recognizes the flagella connector also in fixed non‐detergent‐treated SMOXP9 cells expressing eYFP::FCP4/TbKin15 (green). DNA was visualized with DAPI (blue). D. Micrographs of parental and Δ*FCP4/TbKin15* cytoskeletons stained with mAb62 (magenta) and with DAPI stained DNA (blue). The arrows indicate the flagella connectors with the mAb62 signal and the arrowheads the flagella connectors without the signal. The additional signals from mAb62 are particular noticeable in D) as the contrast has been increased to show that no flagella connector signal remains in the case of Δ*FCP4/TbKin15*.

A screen of monoclonal antibodies raised against detergent resistant cytoskeletons of procyclic trypanosomes identified one, mAb62, which strongly labelled the distal tip of the new flagellum in all 2F cells by immunofluorescence (Fig. 1A). mAb62 also stained the basal body, pro‐basal body, flagellum and the nucleus; however, the new flagellum tip signal was the predominate one (Fig. 1D). Intriguingly, in addition to 2F cells the strong mAb62 signal was also present at the tip of the flagellum of 10% of 1F1K1N cells (*N* = 22 of 219 cells) (Fig. 1A–C). This pattern in the cell population was highly reminiscent of the behaviour of the recently identified constituent of the new flagellum zone of the FC, FCP4/TbKin15 (Tb927.10.890), which was shown to be also present in N‐FCR (Varga *et al.*, 2017). We therefore performed mAb62 staining on cells in which one allele of FCP4/TbKin15 had been endogenously tagged with eYFP (Varga *et al.*, 2017). The mAb62 signal always co‐localized with the eYFP signal (Fig. 1B and C). However, the two signals often did not completely overlap. FCP4/TbKin15 is a large protein, which consists of nearly 3000 amino acids (Varga *et al.*, 2017). Given the estimated contour length of an amino acid being about 0.4 nm (Ainavarapu *et al.*, 2007), the FCP4/TbKin15 protein may span hundreds of nanometres, especially if stretched by the two beating flagella. The protein was tagged with eYFP at the N terminus, hence the observed offset between the two signals could be caused by mAb62 targeting a region of the protein distal to the N terminus. Alternatively, mAb62 could recognize an epitope distinct to FCP4/TbKin15; however, no FC constituent identified so far, other than FCP4/TbKin15 has been shown to be a constituent of N‐FCR. Western blotting would allow us to distinguish between these two possibilities, but mAb62 does not appear to specifically recognize any protein using this method.

Hence, to further test whether FCP4/TbKin15 is the antigen of mAb62 we performed immunofluorescence using the FCP4/TbKin15 deletion cell line (Δ*FCP4/TbKin15*). In this cell line, an aberrant flagella connection was observed in 7.4% of 2F1K1N cells (*N* = 10 of 136 cells), in 33.3% of 2F2K1N cells (*N* = 12 of 36), in 46.7% of mitotic cells (*N* = 7 of 15), and in 69.6% of post‐mitotic cells (*N* = 16 of 23), which is similar to the previously reported FCP4/TbKin15 RNAi knock down phenotype (Varga *et al.*, 2017). In 2F Δ*FCP4/TbKin15* cells there was no FC‐associated mAb62 signal observed in either cells with flagella connected or disconnected at the FC (*N* = 30 cells each) (Fig. 1D). In accordance, there were no 1F1K1N cells (*N* = 100) observed with the flagellum tip signal. The weak basal body, pro‐basal body, flagellar and nuclear signals were not affected by FCP4/TbKin15 deletion (Fig. 1D). This indicates that FCP4/TbKin15 is either the antigen recognized by mAb62 in the FC or the localization of the antigen to the FC is FCP4/TbKin15 dependent.

### Combination of mAb62 with eYFP::FCP3 distinguishes 1F1K1N cells inheriting either the new or the old flagellum

**Figure 2:**
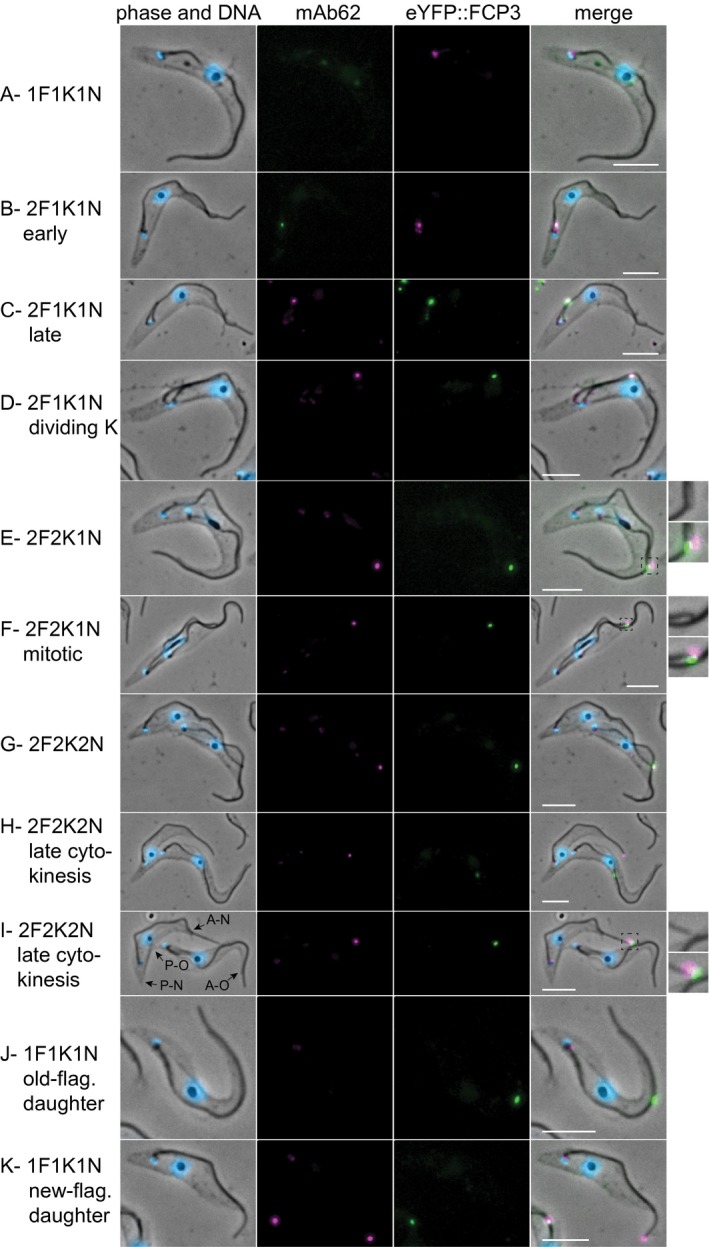
Combination of mAb62 and eYFP::FCP3 enables identification of new‐flagellum daughters and old‐flagellum daughters. Cytoskeletons of cells expressing eYFP::FCP3 (green) stained with mAb62 (magenta) and DAPI (blue) at different stages during the cell cycle. Scale bars represent 5 µm. Insets in 2E, F and I show a higher magnification view of the flagella connector region; in these cells the side view of the flagella connector allows for an unambiguous determination of the position of signals along its new flagellum—old flagellum axis. The cells in 2H and I demonstrate that the flagella connector severing happens at variable points in cytokinesis (Briggs *et al*., 2004). Arrows in 2I indicate the anterior (A–N and A–O) and posterior (P–N and P–O) cell ends of the emerging new‐flagellum and old‐flagellum daughter cells, respectively.

Previously, we identified protein FCP3 (Tb927.8.7540) as part of the old flagellum zone of the FC, and the signal of eYFP‐tagged FCP3 persists post‐cytokinesis approximately halfway along the old flagellum in O‐FCR (Varga *et al.*, 2017). To distinguish both OFDs and NFDs in a single population to examine in detail differences between them we stained the eYFP::FCP3 expressing cell line with mAb62 (Fig. 2). In these cells the eYFP FC‐associated signal was present as soon as the cell started assembly of the new flagellum and was always accompanied by a strong mAb62 signal (Fig. 2B). The two signals completely or partially overlapped (Fig. 2B–I). In the case of a partial overlap the FCP3 signal was closer to the old flagellum than the mAb62 signal (Fig. 2E, F, I), consistent with the localization of FCP3 and FCP4/TbKin15 proteins to the old and new flagellum zones of the FC, respectively (Varga *et al.*, 2017). In cells in cytokinesis with the flagella connection severed (the tip of the new flagellum had separated from the side of the old flagellum), the mAb62 signal was found at the tip of the new flagellum and the eYFP::FCP3 signal approximately half way along the old flagellum (Fig. 2H). This is consistent with 1F1K1N cells with the eYFP::FCP3 or mAb62 signal in their flagellum (Fig. 2J and K) being products of a recent cytokinesis event and representing the OFDs and NFDs, respectively.

1F1K1N cells constitute the largest morphological category of cells present in an exponentially growing culture (Sherwin and Gull, 1989b). In agreement with this, among a total of 515 cells we counted 219 1F1K1N cells of which 22 had a mAb62 signal at the tip of their flagellum and 7 had a eYFP::FCP3 half way along their flagellum. The majority of 1F1K1N cells therefore did not possess a visible marker of flagellar inheritance, indicating a rapid removal of the FCRs post‐cytokinesis. In fact, ergodic analysis (Wheeler, 2015) for this experiment showed that the mAb62 signal was only observed on 1F1K1N cells for the first ~34 minutes and FCP3 for the first ~11 minutes of the cell cycle post cytokinesis. These observations suggest that our double marker system enables us to divide the 1F1K1N cells from a single cell culture into three cohorts: (i) daughters of a recent cytokinesis inheriting the new flagellum (NFDs), (ii) daughters of a recent cytokinesis inheriting the old flagellum (OFDs) and (iii) the cells that underwent cytokinesis at an earlier time (no FCR cells).

### New‐flagellum daughter cells are morphologically distinct to the remaining two cohorts of 1F cells

**Figure 3:**
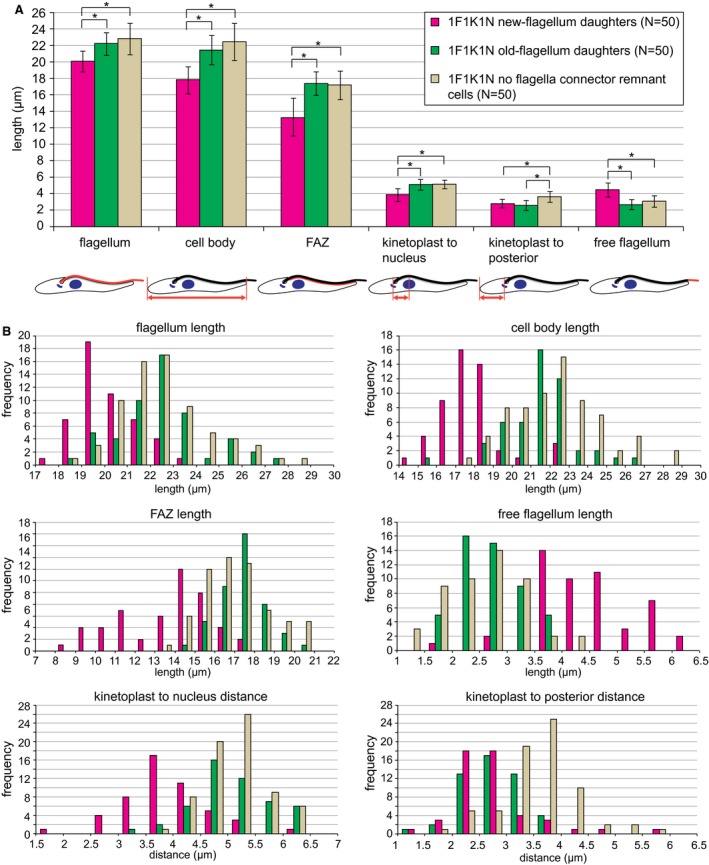
Morphometric analysis of the three cohorts of 1F1K1N cells. Data were obtained on cytoskeletons of cells expressing eYFP::FCP3 and stained with mAb62 and DOT1—for images see Fig. S1. A. Mean lengths/distances (± s.d.) of different cell parameters for the three 1F1K1N cell cohorts. Horizontal bars with stars indicate a significant difference at *P* < 0.05 as determined by *t*‐test. *N* = 50 for each cell cohort. Cartoons below indicate the parameter measured (in red). B. Histograms of different parameters shown in A for the three cohorts of 1F1K1N cells.

To identify if there were any gross morphological differences between these three cell cohorts we measured a series of cell parameters (Fig. 3). We stained cytoskeletons of cells expressing eYFP::FCP3 with mAb62 and DOT1 (Woods *et al.*, 1989), a marker of the FAZ, which is an important morphogenetic structure (Fig. S1) (Kohl *et al.*, 2003; Hayes *et al.*, 2014; Sunter *et al.*, 2015). In addition, the DNA was stained with DAPI highlighting the nucleus and the kinetoplast. The flagellum length, cell body length, DOT1 signal length (FAZ), distance between the kinetoplast and nucleus, distance between the kinetoplast and the posterior end of the cell and free flagellum length were measured for 50 1F1K1N cytoskeletons of each cohort (NFDs, OFDs, no FCRs) (Fig. 3). These measurements revealed that the NFDs had shorter flagellum, cell body, FAZ and kinetoplast to nucleus distance than the OFDs and no FCR cells. The NFDs did, however, possess a longer free flagellum (Fig. 3).

In our measurements there were no morphological differences between the OFDs and 1F1K1N no FCR cells apart from an increase in the kinetoplast to the posterior cell end distance. This increase is likely due to the no FCR cells being further through the cell cycle and therefore beginning to extend the posterior end of the cell.

### Identification of the new‐ and old‐flagellum daughter cells based solely on antibodies

The antibody, MPM2, which recognises phosphorylated serine and threonine residues has previously been shown to label the FC as well as other cytoskeletal structures (Davidge *et al.*, 2006). In addition to 2F cells, a subset (~13%) of 1F1K1N cells possessed a MPM2 signal half way along their flagellum, likely marking the O‐FCR (Davidge *et al.*, 2006). Both MPM2 and mAb62 are mouse IgG antibodies making it difficult to differentiate between their signals. Hence, to create a flexible antibody‐only approach for identification of OFDs and NFDs we generated a mouse monoclonal IgM antibody, mAb35C that recognises the FC and the N‐FCR (Fig. S2). The immunofluorescence staining pattern of mAb35C is relatively complex as it recognises the nucleus and FAZ in addition to the FC (Fig. S2). However, only the FC and FCR‐associated signals of mAb35C are absent in Δ*FCP4/TbKin15* cells (Fig. S2A).

This antibody‐only approach allowed us to study how universal are the morphological differences between NFDs and OFDs. We analysed cultures of SMOXP9 cells (a TREU 927‐based cell line) and 29:13 cells (a Lister 427‐based cell line) (Wirtz *et al.*, 1999; Poon *et al.*, 2012) (Fig. S2B and D). The results were similar to mAb62/eYFP::FCP3 experiments; in all cases, the NFDs had shorter flagellum, cell body, FAZ and kinetoplast to nucleus distance but a longer free flagellum than the OFDs (Fig. S2C and E).

### Morphological re‐modelling in the new‐flagellum daughter cells through the cell cycle

**Figure 4:**
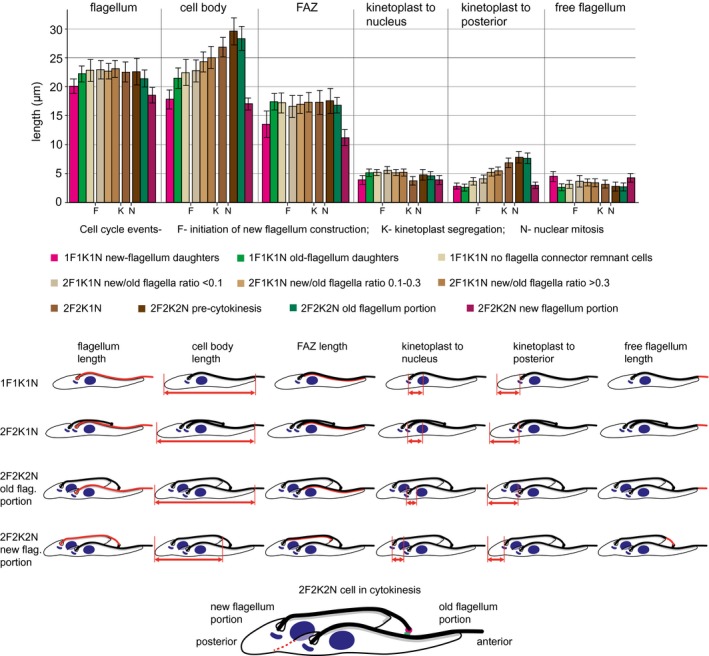
Morphometric analysis of cytoskeletons of different cell cycle stages. Cells expressing eYFP::FCP3 were stained with mAb62 and DOT1—for images see Fig. S1. Mean lengths/distances (± s.d.) of different cell parameters for different cell cycle stages were plotted. Timing of major cell cycle events, such as initiation of new flagellum construction, kinetoplast segregation and nuclear mitosis are indicated. *N* = 24 for 2F2K2N old and new flagellum portions and *N* = 50 for the remaining categories. The data for the three cohorts of 1F1K1N cells are reproduced from Fig. 3. Cartoons indicate parameters measured for different cell cycle stages (in red). For 2F1K1N cells the parameters were measured in the same way as for 2F2K1N cells. Note that in the case of the old flagellum portion of 2F2K2N cells the whole body length was measured; the posterior cell end of the old‐flagellum daughter is not yet readily discernible. The cartoon at the bottom indicates the new and old flagellum portion of a 2K2N cell in cytokinesis. The red dashed line indicates how the cell will be resolved by the cytokinetic furrow into two daughters.

It was shown previously that the NFD flagellum grows post cytokinesis (Farr and Gull, 2009). In order to investigate whether the other NFD‐specific features undergo changes as part of NFD morphological transformation post cytokinesis we extended our morphometric analysis to the entire cell cycle (Fig. 4). During the cell cycle, procyclic forms progress through readily definable cell configurations—1F1K1N, 2F1K1N, 2F2K1N followed by 2F2K2N before and during cytokinesis. Moreover, to provide greater temporal resolution within the relatively large 2F1K1N category we took advantage of the well‐established relationship between the new flagellum length and the cell cycle status of a cell (Robinson *et al.*, 1995) and further sub‐divided the cells into three cohorts with new/old flagellum length ratio of (i) <0.1, (ii) 0.1–0.3 and (iii) >0.3 (Fig. 4). For the morphometric parameters we measured the OFDs were very similar to the old flagellum portion of the 2F2K2N cytokinetic cell (i.e. the part of the 2F2K2N cell body associated with the old flagellum). In contrast, NFDs had a longer cell body, flagellum and FAZ than the new flagellum portion of 2F2K2N cells in cytokinesis (Fig. 4), implying that the nascent NFD continues to grow through cytokinesis; however, this growth occurs without altering the kinetoplast to nucleus and kinetoplast to posterior cell end distances (Fig. 4).

Our extensive analysis shows that the 1F1K1N NFDs have a distinct morphology from that of the 1F1K1N OFDs or 1F1K1N no FCR cells and those cells in later cell cycle stages. This suggests that the re‐modelling of NFD cell morphology occurs rapidly and early in the cell cycle.

### Re‐modelling of the anterior end of the new‐flagellum daughter cells

**Figure 5:**
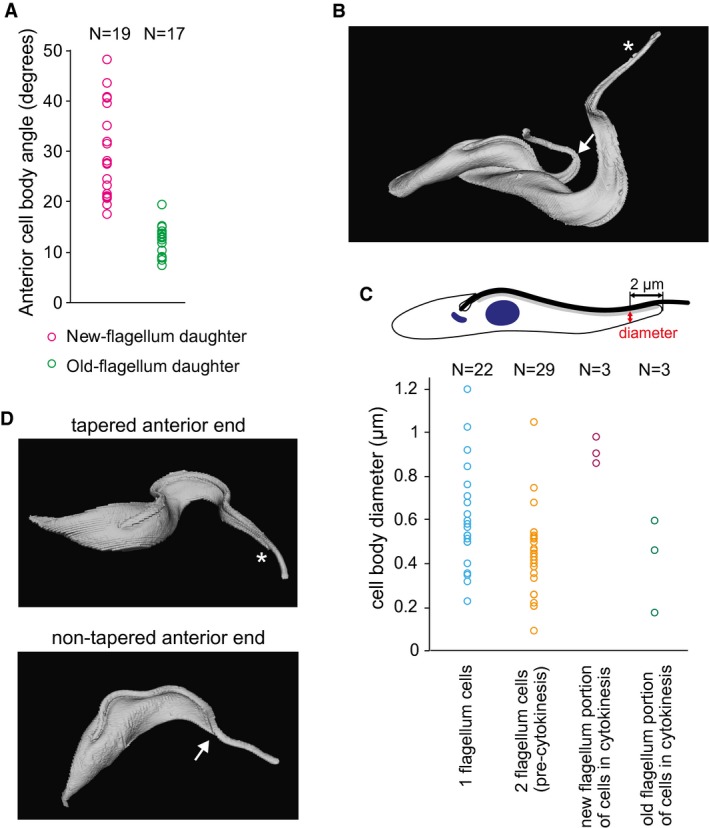
Re‐modelling of the anterior cell end. A. Measurement of the angle at which the cell body meets the flagellum in new‐ and old‐flagellum daughter cells in phase contrast images of fixed cells. B. Example SBF‐SEM acquired model of a cell in cytokinesis with the tapered end of the old flagellum portion of the cell indicated with an asterisk and the non‐tapered end of the new flagellum portion indicated with an arrow. C. Measurement of the radius of the cell body 2 µm from the anterior cell end for 1F cells, 2F pre‐cytokinesis cells and for the new flagellum and old flagellum portion of cells in cytokinesis that were modelled using the SBF‐SEM data. A cartoon at the top highlights the position at which the measurement was taken. D. Example models of 1F cells built from the SBF‐SEM data highlighting the tapered (an asterisk) and non‐tapered (an arrow) anterior cell ends.

In a previous study, we described a difference in the shape of the posterior end between the two types of daughter cells, with the NFDs having a rounder end and the OFD having a pointed end (Wheeler *et al.*, 2013), which we also observed in this study (see Fig. 2J and K). Here, we in addition report that a difference in shape to the anterior end of the cells also exists. Analysing phase contrast images of whole cells we noticed that in OFD cells the cell body had a tapered anterior cell end, which met the flagellum at a relatively shallow angle of 12.3 ± 3.0 degrees (mean ± s.d., *n* = 17) (Figs 2J and 5A). In NFD cells, the cell body had a non‐tapered anterior, which met the flagellum at a steeper angle of 29.5 ± 9.3 degrees (*n* = 19) (Figs 2K and 5A).

To investigate this phenomenon further we analysed 58 cells of an asynchronous cell culture by serial block face‐scanning electron microscopy (SBF‐SEM). As it is not possible to use molecular markers to distinguish different cohorts of 1F cells with this technique we first focused on the cells in cytokinesis, whose new and old flagellum portion resemble the NFD and OFD cells, respectively (Fig. 4). We observed that the new flagellum portion had indeed a non‐tapered anterior end, whereas the old flagellum portion had a tapered anterior end (*N* = 3) (Fig. 5B). To quantify this further, we measured the diameter of the cell body 2 µm from the anterior end (Fig. 5C) and observed that it was indeed significantly larger in the case of the new flagellum portion (908 ± 58 nm vs. 403 ± 215 nm, Fig. 5C). For 1F cells, the diameter of the anterior cell end was spread over a wide range encompassing both the large diameters observed for the anterior end of the new flagellum portion as well as the small diameters of the old flagellum portion of cells in cytokinesis (Fig. 5C, examples of the two types of 1F cells shown in Fig. 5D). The diameter of the anterior end of 2F cells before cytokinesis, including cells with short new flagella, had a narrower distribution, which more closely matched that observed for the anterior end of the old flagellum portion of cells in cytokinesis (Fig. 5C). These data clearly demonstrate that the re‐modelling of a NFD cell includes architectural change in the anterior end of the cell body in G1 of the cell cycle.

Whilst analysing the images of cytoskeletons stained with the DOT1 antibody we observed that on a fraction of 1F1K1N cells the DOT1 signal extended from the anterior end of the cell on the opposite side of the cell body to the FAZ for ~1 µm (in addition to the regular DOT1 FAZ signal present in all cells) (Fig. S3). This fish hook barb like ‘V’‐shaped signal of DOT1 was present exclusively in the cohort of the NFD cells and was observed in 36% (*N* = 18 of 50) of them. This is a likely underestimate since, depending on how cells settled onto the slide before imaging, the signal could have been obstructed in some cells by the FAZ signal. The ‘V’‐shaped signal is likely related to the re‐modelling that occurs to the anterior end of the NFD cells during the transition from a non‐tapered to a tapered anterior cell end morphology.

### Duration of G1 is not affected by the cell re‐modelling

**Figure 6:**
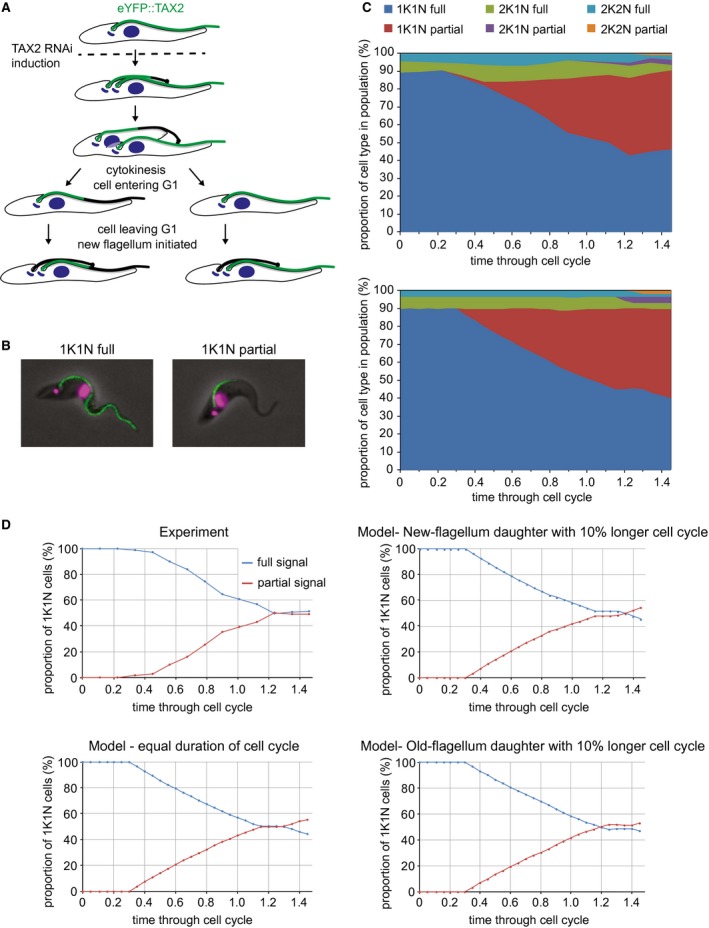
G1 phase duration is equal in both daughter cells. A. Schematic showing the types of eYFP::TAX2 expressing cells generated after induction of RNAi against TAX‐2. The first round of new flagella produced after RNAi induction will have eYFP signal in the proximal part of the flagellum, flagella produced thereafter will have no eYFP signal. B. Micrographs showing an example cell with eYFP::TAX2 signal along the full length of its flagellum (left) and a cell with eYFP::TAX2 signal in the proximal part of the flagellum (right). DNA stained with DAPI is shown in magenta. C. Plots comparing the modelled and experimental results following induction of RNAi. Cells were categorized by cell cycle stage and flagellar signal type. Initially all cells have a full signal flagellum (blue, green, turquoise), then cells appear with partially labelled flagella, first in the 1K1N category (red) followed by 2K1N (purple) and 2K2N (orange). Partially labelled flagella are slower to appear in the observed results than in the model. *N* = 500 cells at each timepoint. D. To determine relative duration of G1 phase of the cell cycle of old‐flagellum versus new‐flagellum daughter cells, the value at which the proportion of each type within the 1K1N population reaches a plateau is critical. Experimental results show that this occurs at 50% for both types; this is the same as in the modelled results where all cells were dividing at the same rate. To characterize the sensitivity of our approach, we also modelled how would proportions of both cell types change if the new‐flagellum daughter cell cycle lasted 10% longer; in that case the plateau would be reached at 48% partially labelled and 52% fully labelled flagella. If the old‐flagellum daughter cell cycle lasted 10% longer the proportions would be reversed.

Our data show that significant morphological changes are associated in particular with maturation of the NFD cell, which occur in the G1 phase of the cell cycle, by the time the cell starts to construct a second flagellum. We asked whether these changes have an impact on the duration of the G1 phase. We analysed a cell line containing an inducible RNAi construct targeted to the axonemal protein, TAX‐2, one allele of which was tagged with eYFP (Farr and Gull, 2009). This cell line provides a way of following for longer periods the NFD and OFD cells. A flagellum made before RNAi induction will have eYFP signal along the full length of its axoneme, whereas a new flagellum that was constructed whilst RNAi took effect will be missing eYFP signal in its distal region assembled after the tagged protein was depleted; hence the flagellum will be partially labelled (Fig. 6A and B). Eventually, cells that started flagellum assembly after RNAi took effect will appear in the population; these will be completely devoid of the eYFP signal. Knock down of TAX‐2 induced for 72 h was shown to cause a variety of axonemal defects in 16% of cells, including both a decrease and an increase in the number of microtubule doublets (Farr and Gull, 2009). However, as these defects had no effect on population growth rate (Farr and Gull, 2009), and given our experiment occurred over a much shorter time span we did not expect the presence of these mutants to affect our subsequent analysis and we, therefore, did not account for them.

A mathematical model allowed us to explore how the proportions of cells in a population with these different flagellar signal types will change dependent on the duration of their cell cycle (Fig 6C and D). One of the most important points predicted by the model is that the proportion of 1K1N cells with an eYFP signal along the full length of the axoneme in a population will reach a plateau. This plateau is reached when all the cells with a flagellum made pre‐RNAi induction have completed a cell cycle generating one 1K1N cell with eYFP signal along the entire flagellum and another 1K1N cell with a partially labelled flagellum (Fig. 6A). The model predicted that if the OFD and NFD cells divide at the same rate, the proportion within the 1K1N population will be equal (Fig. 6D). However, if one or other divides faster, that category would be proportionally overrepresented in the population (Fig. 6D).

We then performed a time course experiment, in which cells were fixed at intervals from 0 to 13 h post TAX2 RNAi induction. These samples were examined by fluorescence microscopy to determine both the cell cycle stage from DAPI signal and the eYFP signal in the flagellum (Fig. 6C and D). In these experimental data, the proportion of old‐flagellum 1K1N cells (with the eYFP signal along the entire flagellum) reaches a plateau between 1.24 and 1.46 cell cycle units post‐induction (with a cell cycle unit being equal to 8.9 h this corresponds to 11 and 13 h) at 50% of 1K1N cells, equal to the proportion of new‐flagellum 1K1N cells (partial or devoid of eYFP signal) (Fig. 6D). This result indicates that despite each of the daughters undergoing re‐modelling of distinct cytoskeletal structures in the G1 phase, duration of this phase in both daughters is equal.

## Discussion

Here, we took advantage of the asymmetric nature of the FC and its resolution to identify trypanosome 1F1K1N cells inheriting either the old or the new flagellum post cytokinesis. We have termed these respectively OFD and NFD cells.

**Figure 7:**
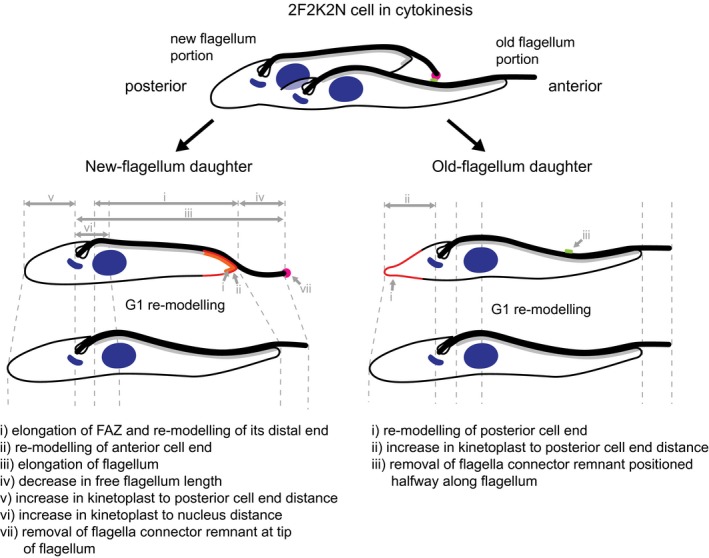
Schematics of changes to the new‐flagellum and old‐flagellum daughters in G1. Major changes to morphology of new‐flagellum daughter cells and old‐flagellum daughter cells detected in this study and in Wheeler *et al*. (2013) during G1 phase are indicated with arrows. Areas of major cytoskeletal re‐modelling are highlighted in red. See Fig. 4 for measurements.

As these NFDs and OFDs progress through the G1 phase they re‐model in two distinct and highly defined ways to produce the canonical morphology of 1F1K1N cells in the later G1 of the cell cycle. By the time these cells start constructing a second flagellum in S phase (Woodward and Gull, 1990), they are all morphologically similar (Fig. 4). Re‐modelling of the OFD cells involves rounding of the posterior cell end (Wheeler *et al.*, 2013) and the removal of the O‐FCR. Re‐modelling of the NFD cells is more extensive and includes elongation of the cell body, flagellum and FAZ, reduction in length of the free portion of the flagellum, transition to a tapered anterior cell end morphology, and the removal of the N‐FCR (Fig. 7).

We believe the pattern of NFD and OFD cell end re‐modelling is related to the asymmetric nature of the trypanosome cytokinesis process, which results in each daughter cell inheriting different parts of a dividing cell. While both daughters share the middle zone, the NFD only inherits the posterior zone, and the anterior zone is inherited entirely by the OFD (Wheeler *et al.*, 2013). As these zones have been formed before cytokinesis they do not require re‐modelling. The unique morphological features specific to each of the nascent daughter cells, namely the non‐tapered anterior cell end of the NFD and the pointed end of the OFD, are on the other hand formed during cytokinesis. Thus, we assume they represent a constraint on cell morphology imposed by the cytokinesis process that has to operate without the disassembly of the interphase microtubule‐based cellular and flagellar cytoskeleton. As such these features can perhaps be compared to the bud and birth scar of budding yeast, the well‐studied microbial model of asymmetric proliferative division. Trypanosome cells, however, are able to lose these juvenile features rapidly, as their shape is determined by the microtubule‐based cytoskeleton, highly amenable to re‐modelling.

A procyclic cell undergoes the NFD type of re‐modelling only once in its life, namely in the first cell cycle following the one in which its flagellum (and other conservatively inherited structures such as the FAZ) were assembled; this is analogous to the maturation of the centriole, which also happens in the cell cycle following the one in which it was formed (Nigg and Stearns, 2011). The OFD type of re‐modelling is on the other hand performed periodically in every subsequent cell cycle. Each procyclic cell is thus capable of two distinct types of re‐modelling of its cytoskeletal structures as part of its G1 phase, and the decision which of them is utilized is determined by the cell's history.

If one assumes that procyclic trypanosome cells do not age rapidly, i.e. a cell is capable of undergoing numerous cell divisions without a significant slow‐down, the ratio of 1F cells in culture inheriting either an old or new flagellum in the last cell division should be 1:1 and this is consistent with our analysis of the TAX2 tagged cell line (Fig. 6). The 1:1 ratio means that for the 1F no FCR cells a bimodal distribution for parameters like FAZ length and flagellum length might be expected; however, no such bimodal distribution was observed (Fig. 3). In fact, the distribution of morphometric parameters for 1F no FCR cells was similar to the OFD cells (Fig. 3), indicating that re‐modelling of the NFD cells post cytokinesis happens rapidly at the beginning of G1. This is also true for removal of both N‐ and O‐FCRs.

One interesting implication of the trypanosome mode of cytoskeleton inheritance is that a cell retains the anterior zone of its cytoskeleton and the flagellum for its entire life. The duration of the lifespan of a procyclic cell has to our knowledge not been experimentally determined, but has to span several cell cycles, as growth of a procyclic culture is in general logarithmic. Given that the rate of turnover of the axonemal constituents was shown to be very low (Vincensini *et al.*, 2018), individual axonemal proteins must withstand mechanical stress linked to the flagellar motility for a considerable length of time.

### Potential mechanisms of morphological re‐modelling

In procyclic cells, a causative link between flagellum length, FAZ length and cell size was demonstrated (Kohl *et al.*, 2003; Zhou *et al.*, 2011; Hayes *et al.*, 2014). Intriguingly, in this work we report that during G1 re‐modelling of NFDs the increase of the FAZ length (13.2 to 17.2 µm) is not matched by that of the flagellum (20.1 to 22.8 µm), and hence is compensated for by a decrease in free flagellum length (Figs 3 and 4). This means that after cytokinesis the FAZ is assembled more rapidly than the flagellum, which is the inverse to what is observed before cytokinesis (Kohl *et al.*, 1999), showing a greater level of flexibility in assembly of the FAZ/flagellum structure than previously thought.

For cell body length to increase microtubules of the sub‐pellicular corset are extended. In 1F1K1N cells new tubulin polymerization has been shown to predominantly occur at the posterior end of the cell, where the microtubule plus ends are located (Sherwin and Gull, 1989a; Wheeler *et al.*, 2013). NFDs are ~5 µm shorter than 1F1K1N no FCR cells; however, a comparatively small increase (of less than 2 µm) in the distance between the kinetoplast and the posterior end of the cell has been observed. This result suggests that either microtubule growth is more prevalent at sites other than the posterior end of the cell in the re‐modelling cells, or that a mechanism for sliding of sub‐pellicular microtubules relative to the FAZ/basal body/kinetoplast unit exists. Related to this is the observed extensive re‐modelling of the cytoskeleton at the anterior end of the NFD cell. The initial non‐tapered shape is defined by the cytokinesis furrow ingression. During re‐modelling it becomes more tapered (Fig. 5), resulting in a more symmetrical shape of the leading edge of the cell; the cell would thus avoid an asymmetric movement path arising from the net effect of propulsive and drag forces, which is likely to preclude directional swimming. This anterior cell end re‐modelling is accompanied by rapid changes in the localization pattern of DOT1, a marker of the FAZ, from the one bending around the anterior cell body end to an essentially straight line following the flagellum. Interestingly, in the bloodstream form of *T. brucei* DOT1 in addition to the linear FAZ signal along the flagellum recognizes an elaboration at the distal end of the FAZ called the groove, which is an indentation of the cell body membrane surrounding the tip of the new flagellum. The groove resolves before cytokinesis with each daughter cell inheriting a linear FAZ (Hughes *et al.*, 2013).

### Applications for new cell cycle markers

Many analyses of phenotypes in trypanosomes rely on examining changes in the morphologies of various cell types as categorized based on kinetoplast and nucleus configurations. This approach results in 1F1K1N cells being treated as a single category. As a cell spends the first half of its cell cycle in the 1F1K1N configuration this relatively simple analysis technique lacks temporal resolution in this substantial part of the cell cycle. Here, we have developed a new set of reagents allowing us to distinguish three cohorts of the 1F1K1N cells. We have shown that the two daughter cells generated during procyclic cell division can be defined using FC proteins that segregate either to the old or new flagellum when the FC is severed. The N‐ and O‐FCR signal was very short‐lived as it was lost within the first 30–40 minutes of the cell cycle. Hence, it gives a precise snapshot of the cells right at the beginning of G1. This allows discerning even subtle changes to morphology of 1F1K1N cells as they progress through the cell cycle, such as the increase in the kinetoplast to the posterior cell end distance (Fig. 3). It should also be noted that as the removal of proteins from the N‐ and O‐FCR is likely to be gradual, the length of the period for which it is possible to detect FCR cells will depend on sensitivity of detection of FCR constituents.

Each of the three cohorts of 1F1K1N cells is morphologically more uniform than 1F1K1N cell category in general. This allows to identify statistically significant morphological differences while taking measurements of a lower number of cells. Moreover, the ability to distinguish the NFD and OFD cells allows studying processes specific to each of them. In summary, these new tools will be of great use when studying a range of mutant phenotypes.

Finally, we believe that due to the mode of cytoskeletal and organelle inheritance operating in these organisms it is likely all proliferative divisions in trypanosomes will generate non‐equivalent daughters.

## Experimental procedures

### Cell lines and cell cultures

*T. brucei* procyclic cells were grown at 28°C in SDM‐79 (Gibco) with 10% FCS (Brun and Schönenberger, 1979). The cultures were maintained between 1 × 10^5^ and 1 × 10^7^ cells ml^−1^ with cell densities measured using the CASY Cell Counter. Cell lines used in the study include SMOXP9 (Poon *et al.*, 2012) SMOXP9 eYFP::FPC3 and SMOXP9 eYFP::FPC4/TbKin15 (Varga *et al.*, 2017), 29:13 eYFP::FLAM3 (this cell line was used as an example of a 427 procyclic cell line and was derived from the commonly used tetracycline inducible 29:13 cell line [Wirtz *et al.*, 1999; Sunter *et al.*, 2015]), 29:13 eYFP::TAX2 and TAX2 RNAi (Farr and Gull, 2009). To generate the Δ*FCP4/TbKin15* cell line the sequence targeting the region immediately upstream of the Tb927.10.890 ORF was amplified by PCR using primers ACTGGGATCCGTGCACCATCTTAAGTTGCT (containing a BamHI restriction site) and CAGTCATATGTTCTTCCTCCTGTGATTCTACT (containing a NdeI restriction site), and the region immediately downstream of the Tb927.10.890 ORF was amplified using primers ACTGTTCGAACAGAAAAGGATGCACTTGTCG (containing a BstbI restriction site) and CAGTGAGCTCTCACTGCTTACTTTC (containing a SacI restriction site). Both PCR products were ligated into plasmids pJ1014 and pJ1015 (Varga *et al.*, 2017). To delete a single allele of the gene, the pJ1014 vector was digested with BamHI and SacI and the fragment containing the targeting sequences and a blasticidin resistance gene was electroporated into SMOXP9 cells following a standard protocol (McCulloch *et al.*, 2004). Following drug selection positive clones were obtained and used for deletion of the second allele with the pJ1015 vector conferring G418 resistance.

### Preparation of mAb62 antibody

Detergent‐insoluble flagellar cytoskeletons of cells expressing SAS6::GFP (Tb927.9.10550) and with RNAi against kinesin II (Tb927.11.13920) induced for 5 days were prepared following the 65 mM CaCl_2_ protocol (Sunter *et al.*, 2015). Protein amount was quantified using a BCA assay. Sample aliquots of 0.8 mg protein were kept at −80°C until use. Balb/C mice were immunized with 0.25 mg protein each, (i.p.) in emulsified immunogens of Freund's complete adjuvant, following three boosts of protein in emulsified immunogens of Freund's incomplete adjuvant at two week intervals. Mice were sacrificed on the fourth day after final boost, and splenocytes were collected using 0.1 mm pore filter and fused to SP/2.0 myeloma cells (Woods *et al.*, 1989). Positive clones were selected with HAT medium. Neat supernatant from the individual wells was screened by immunofluorescence. mAb62 expressing cells were cloned by three rounds of limiting dilution cloning. All procedures were performed under Home Office Licence and in accordance with Institutionally‐approved protocols.

### Preparation of the monoclonal mAb35C antibody

Detergent‐insoluble flagellar cytoskeletons were prepared from 427 procyclic cells following the 65 mM CaCl_2_ protocol (Sunter *et al.*, 2015). The fraction containing purified flagellar cytoskeletons was acetone precipitated, ground using a mortar and pestle, and dissolved in PBS. Immunisation of mice, splenectomy, fusion of splenocytes with myeloma cells, and cloning of resulting hybridoma cells was performed by OxFabs. Hybridoma cell lines were screened for the presence of antibodies recognizing the new flagellum tip by immunofluorescence on methanol‐fixed detergent‐resistant trypanosome cytoskeletons.

### Immunofluorescence

Cells were grown to densities of ~5 × 10^6^ cells ml^−1^, washed with PBS, resuspended to ~2 × 10^7^ cells ml^−1^ in PBS, and settled on microscope slides. Subsequently, they were either fixed in −20°C methanol or processed for preparation of cytoskeletons by incubation in PEME (100 mM PIPES pH 6.9, 1 mM MgSO_4_, 2 mM EGTA, 0.1 mM EDTA) + 0.5% Igepal CA‐630 (v/v) for 30 s. The cytoskeletons were then fixed in −20°C methanol for 20 min. After fixation both whole cells and cytoskeletons were rehydrated in PBS for 20 minutes at room temperature followed by blocking with PBS+1% BSA for an hour. Primary antibodies (mAb62 (IgG) 1:200, DOT1 (IgM) neat (Woods *et al.*, 1989), mAb35C (IgM) 1:10, MPM (IgG) 1:200) were diluted in blocking buffer and applied for 1 h. The samples were washed thoroughly in PBS before the secondary antibody was added (1:200) in blocking buffer for 1 h. The samples were washed 3 × 5 minutes in PBS and mounted in 90% glycerol with 1,4‐diazabicyclo[2.2.2]octane and 100 ng ml^−1^ of 4′,6‐diamidino‐2‐phenylindole (DAPI).

### Microscopy and image analysis

Phase contrast and fluorescence images of fixed whole cells and cytoskeletons were acquired using a Leica DM5500B microscope with a Leica 100× (NA 1.4) HCX PL APO oil immersion objective and an Andor Neo 5.5 sCMOS camera, or with a Zeiss Axioplan 2 microscope with a Zeiss 100x (NA 1.4) Plan Apochromat oil immersion objective and an Andor Zyla 4.2 camera. The images were acquired using Micromanager (Edelstein *et al.*, 2010). Morphometric measurements were performed using ImageJ software (Schneider *et al.*, 2012) (National Institutes of Health, Bethesda, Maryland, USA).

### Serial block‐face scanning electron microscopy

For this work, we reanalysed the SBF‐SEM data collected previously for the analysis of TAC65 RNAi cells (Hoffmann *et al.*, 2018). Uninduced TAC65 RNAi cells were harvested, resuspended in the same medium, and fixed in culture using 2.5% glutaraldehyde. After initial fixation, cell pellets were resuspended in 100 mM phosphate buffer pH 7.0, containing 2.5% (w/v) glutaraldehyde, 2% (w/v) paraformaldehyde, and 0.1% tannic acid. Pellets were washed with 100 mM phosphate buffer pH 7.0 and then with 200 mM cacodylate buffer pH 7.4 and embedded in 4% (w/v) gelatin and 4% (w/v) low melting agarose in 200 mM cacodylate buffer. Gelatin embedded pellets were post‐fixed in 1% (w/v) osmium tetroxide and 1.5% (w/v) potassium ferrocyanide in 100 mM cacodylate buffer for 1 h. The samples were rinsed and incubated in 2% (w/v) osmium tetroxide for 1.5 h. After rinsing, samples were stained en bloc overnight in 2% (w/v) uranyl acetate. Samples were then rinsed and processed en bloc by Walton's lead aspartate stain for 30 minutes. Samples were then dehydrated in an ascending acetone series and embedded in TAAB 812 hard resin (TAAB Laboratories Equipment Ltd.). Resin‐embedded samples were trimmed and placed into a Zeiss Merlin VP Compact (Zeiss) fitted with a Gatan 3view2XP system (Gatan). Serial images of the block face were recorded at an accelerating voltage of 4 kV, a spot size of 1, and pressure of 0.338 Torr. Pixel size and the dwell time for each micrograph was 3.5 nm, 3.5 μs, and slice thickness was 100 nm. Images were recorded using Digital Micrograph. Images were stacked, smoothened and aligned using IMOD and ETOMO software (University of Colorado).

### SBF‐SEM data modelling

The shapes of 58 cells in a single SBF‐SEM stack were segmented in Microscopy Image Browser. First, a mask was created separating the cells from the background using black and white thresholding. The mask was subsequently refined using the material statistics to remove small non‐cell material and filled to remove holes within the cells. Where cells were touching they were manually separated on each slice using the brush tool. The shapes of individual cell were then segmented using the object picker and each cell shape was assigned to one material. The cell shapes were then rendered in 3dmod. The cell diameter 2 µm away from the anterior end was measured by drawing a 2 µm long contour on the cell surface along the flagellum from the anterior towards the posterior end. The diameter of the cell was measured by expanding a sphere centred on the posterior point of this contour and calculating its radius when the sphere intersected the modelled cell surface opposite.

### Analysis of proportions of eYFP::TAX2 cells in population upon TAX2 RNAi

For growth timing analysis, TAX‐2 RNAi cell culture expressing eYFP::TAX2 was divided into two parts. To one of them doxycycline was added to induce RNAi and cell densities of both induced and uninduced cultures were measured using the CASY Cell Counter at 0, 2, 4, 8, 10, 11, 12 and 13 h. At each timepoint‐induced cells were prepared for microscopy and imaged. Five hundred cells were categorized according to the count of kinetoplasts and nuclei, and by the eYFP signal in the old flagellum.

### Modelling of proportions of eYFP::TAX2 cells in population upon TAX2 RNAi

The predicted distribution of cells in different cell cycle stage with a flagellum either fully eYFP‐labelled or partially/fully unlabelled, the cells were treated as non‐interacting agents. Each cell had four properties: progress through the unit cell cycle (u; for the given cell culture corresponds to 8.9 h), whether the old flagellum is fully eYFP‐labelled, partially eYFP‐labelled or unlabelled, whether any new flagellum is fully labelled, partially labelled or unlabelled and whether the cell inherited the old or new flagellum in the previous division. The progress through the unit cell cycle, an onset of new flagellum growth, kinetoplast division and nucleus division were determined from a sample of cells in logarithmic growth (Woodward and Gull, 1990; Wheeler, 2015). This allows the mapping of number of flagella (F), kinetoplasts (K) and nuclei (N) to progress through the unit cell cycle: 1F1K1N for <0.596 u, 2F1K1N for <0.852 u, 2F2K1N for <0.942 u and 2F2K2N for >0.942 u.

About 10,000 cells were simulated. Initially, each cell was assigned to a random cell cycle progress (between 0 u and 1 u), taking into account the bias towards early cell cycle stages arising from binary fission (Wheeler, 2015). The cells were simulated at a time step of 0.01 u, incrementing the cell cycle progress of each cell by 0.01 u. The effective start of RNAi induction was simulated as 0.3 u. Prior to this time, all new and old flagella were flagged as fully labelled. At the time of induction the new flagellum in all cells >0.596 u (cell with two flagella) were flagged as partially labelled. After the time of induction, the new flagella in all cells passing 0.596 u (the start of new flagellum formation) were flagged as unlabelled. Every 0.05 u, the properties of the population were reported as numbers of 1F1K1N, 2F1K1N, 2F2K1N, or 2F2K2N cells with an old flagellum unaffected by RNAi (fully labelled) or affected by RNAi (partially or unlabelled).

On any cell reaching the end of the cell cycle (1 u), cell division was simulated: The original cell was simulated as the cell inheriting the old flagellum, the cell cycle progress was reset to 0 u and the properties of the new flagellum reset. A new cell was simulated as the cell inheriting the new flagellum, with a cell cycle progress of 0 u and the flagellum inheriting the labelling pattern of the new flagellum of the parent cell.

To simulate differing cell cycle progression rates of the daughter cell inheriting the old or new flagellum the increment of cell cycle progress was adjusted: 0.01 u (old) and 0.01 u (new) for equal cell cycle progression rates, 0.009 u (old) and 0.01 u (new) for 10% slower cell cycle progression of the daughter cell with the old flagellum and 0.01 u (old) and 0.009 u for 10% slower cell cycle progression of the daughter with the new flagellum. A 10% difference in cell cycle progression rates was chosen as it is smaller than the difference observed in yeast in suspension culture (Wheeler, 2015). In addition, as our experimental method is sensitive to a 5% difference (or greater) at *P* < 0.05, the 10% difference in cell cycle progression rates we modelled shows the scale of an effect we would have been able to observe if it occurred in our experimental data. The script is available on request.

## Author contributions

D.‐H.L. and K.G. made monoclonal antibodies. J.D.S., V.V. and H.F. conceived and performed experiments and wrote and edited the manuscript. R.J.W. developed and tested the mathematical model and edited the manuscript. M.A., H.V. and T.M. performed experiments. V.V., R.J.W., S.V. and K.G. secured funding and edited the manuscript.

## Conflict of interest

The authors declare no conflict of interest.

## Data Availability

The data that support the findings of this study are available from the corresponding author upon reasonable request.
