# Supplementary material for: Non‐equivalence in old‐ and new‐flagellum daughter cells of a proliferative division in Trypanosoma brucei
Source: Mol Microbiol. 2019 Jul 25;112(3):1024–40. doi: 10.1111/mmi.14345 (PMC6771564; doi:10.1111/mmi.14345)
Supplement: Supplementary file 1 [file MMI-112-1024-s001.pdf]

Supplementary Material

Non-equivalence in old- and new-flagellum daughter cells of a proliferative division in *Trypanosoma brucei.*

Running title: Non-equivalence of *Trypanosoma brucei* daughters

Movin Abeywickrema, Hana Vachova, Helen Farr, Timm Mohr, Richard J. Wheeler, De-Hua Lai, Sue Vaughan, Keith Gull, Jack D. Sunter*, Vladimir Varga*

* J.D.S and V.V. should be considered as joint corresponding and senior authors

jsunter@brookes.ac.uk ; 0044 1865 484409

vladimir.varga@img.cas.cz ; 00420 296442155

Contact information:

Vladimir Varga, Hana Vachova

Institute of Molecular Genetics of the Czech Academy of Sciences, Vídeňská 1083, Prague, Czech Republic

Jack D. Sunter, Timm Mohr, Sue Vaughan

Department of Biological and Medical Sciences, Oxford Brookes University, Oxford, OX3 0BP, UK

Movin Abeywickrema, Helen Farr, Keith Gull

Sir William Dunn School of Pathology, University of Oxford, Oxford, OX1 3RE, UK

Richard J. Wheeler

Peter Medawar Building for Pathogen Research, Nuffield Department of Medicine, University of Oxford, Oxford, OX1 3SY, UK

De-Hua Lai

Center for Parasitic Organisms, State Key Laboratory of Biocontrol, School of Life Sciences, Sun Yat-Sen University, Guangzhou 510275, P.R. China

Keywords: *Trypanosoma brucei*; Protozoan parasite; Cell division; Cytokinesis; Flagellum

Supplementary figure legends

Figure S1. Micrographs of cytoskeletons of cells expressing eYFP:FCP3 stained with mAb62 and DOT1 at different stages of the cell cycle. DNA is stained with DAPI (blue), the monoclonal antibodies mAb62 and DOT1 are in magneta and eYFP:FCP3 is green. Scale bars represent 5 µm.

Figure S2. Combination of MPM2 and mAb35C mAbs enables identification of the old- or new-flagellum daughter cells. Scale bars represent 5 µm.

A) Micrographs of mAb35C staining (magenta) on SMOxP9 and ΔFCP4/TbKin15 cells. The arrow indicates the flagella connector with the mAb35C signal and the arrowheads the flagella connectors without the signal. DNA is stained with DAPI (blue).

B) Micrographs of SMOxP9 cells stained with MPM2 (green), mAb35C (magenta) and DAPI to mark the DNA (blue). Note that the cell line was expressing eYFP::FCP3, however, exposure time and strength of antibody signal means that eYFP::FCP3 signal cannot be seen. The arrow indicates the mAb35C-stained flagella connector remnant in the new-flagellum daughter cell (top) and the MPM2-stained flagella connector remnant in the old-flagellum daughter cell (bottom).

C) Measurement of cell parameters for SMOxP9 1F1K1N new- (N = 19) or old-flagellum (N = 15) daughters. Mean lengths are plotted with the error bars representing the standard deviation.

D) Micrographs of 29:13 cell line stained with MPM2 (green), mAb35C (magenta) and DAPI to mark the DNA (blue). Note that the cell line was expressing eYFP::FLAM3, however, exposure time and strength of antibody signal means that eYFP::FLAM3 signal cannot be seen. The arrow indicates the mAb35C-stained flagella connector remnant in the new-flagellum daughter cell (top) and the MPM2-stained flagella connector remnant in the old-flagellum daughter cell (bottom).

E) Measurement of cell parameters for 29:13 1F1K1N new- or old-flagellum daughters (N = 50 for each). Mean lengths are plotted with the error bars representing the standard deviation.

Figure S3. A fraction of new-flagellum daughter cells possess a 'V' shaped DOT1 signal at the anterior cell end.

Micrographs of 1F1K1N cytoskeletons stained with DOT1 and mAb62 (magenta) that have inherited the new flagellum with and without the DOT1 'V' shaped signal at the anterior cell end. The 'V' shaped signal is indicated with an arrow. DNA is stained with DAPI (blue). Scale bars represent 5 µm.

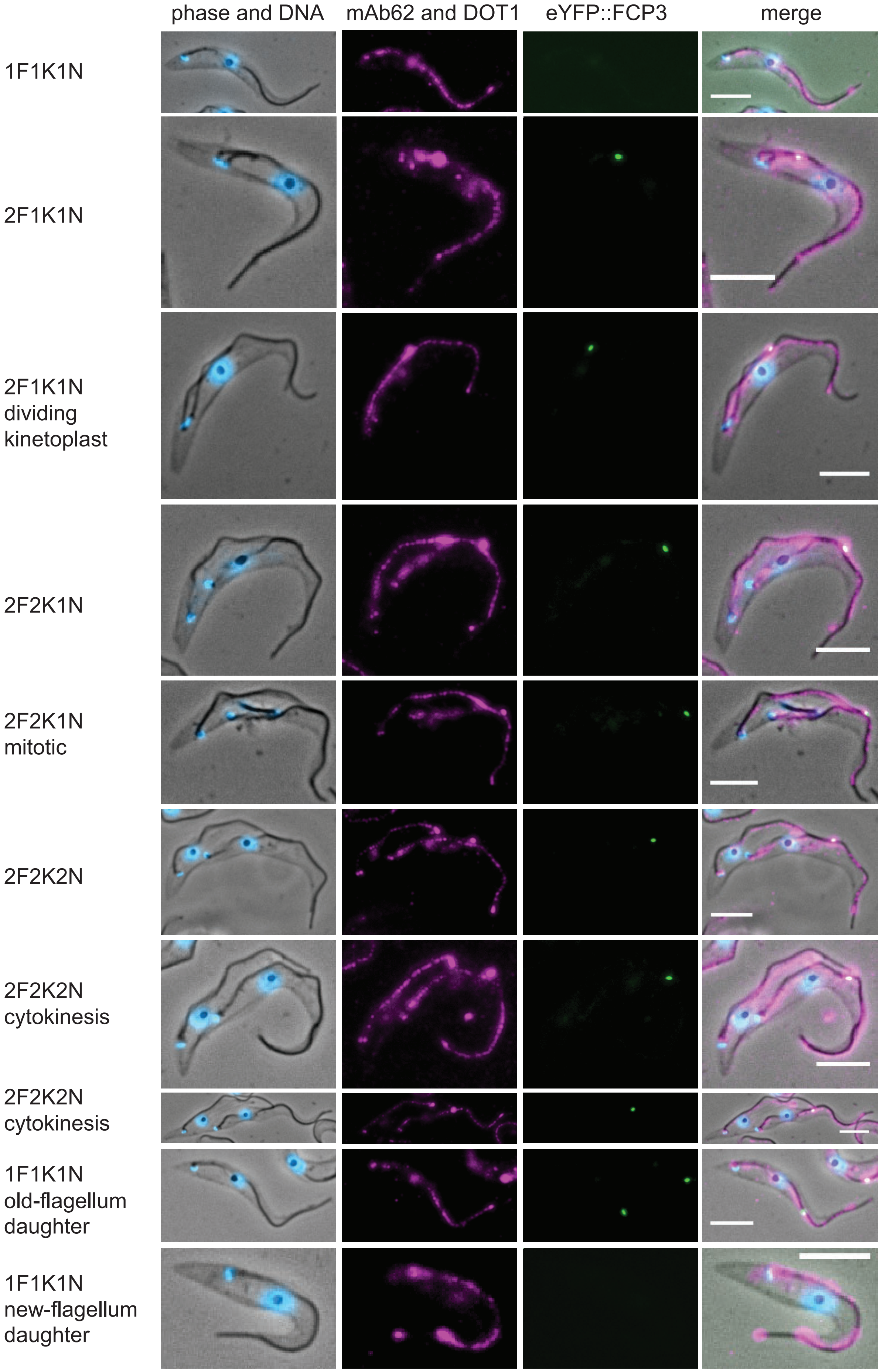

Figure S1. Micrographs of cytoskeletons stained with mAb62 and DOT1 at different stages of the cell cycle.

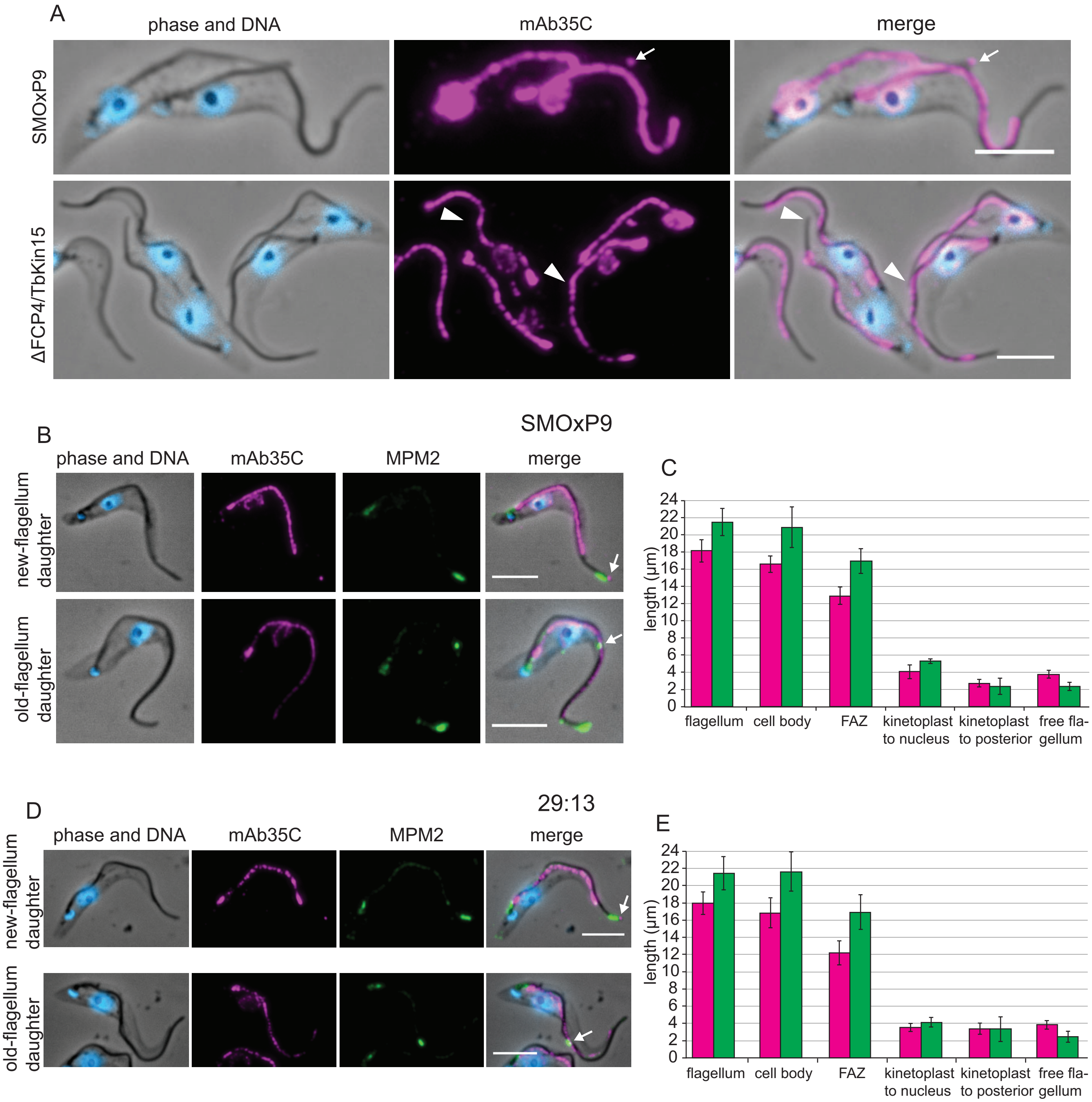

Figure S2 . Combination of MPM2 and mAb35C mAbs enables identification of the old- and new-flagellum daughter cells.

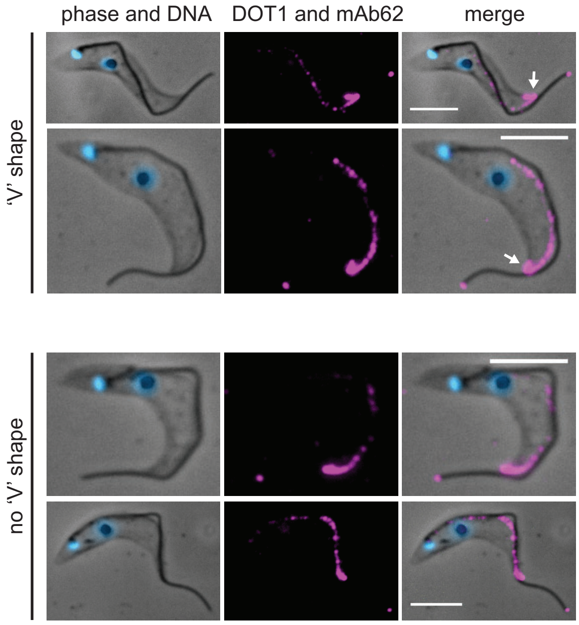

Figure S3. A fraction of new-flagellum daughter cells possess a 'V' shaped DOT1 signal at the anterior cell end.
